# Health after Legionnaires' disease: A description of hospitalizations up to 5 years after *Legionella* pneumonia

**DOI:** 10.1371/journal.pone.0245262

**Published:** 2021-01-11

**Authors:** Shantini D. Gamage, Natasha Ross, Stephen M. Kralovic, Loretta A. Simbartl, Gary A. Roselle, Ruth L. Berkelman, Allison T. Chamberlain

**Affiliations:** 1 National Infectious Diseases Service, Specialty Care Services, Veterans Health Administration, US Department of Veterans Affairs, Washington, District of Columbia, United States of America; 2 Division of Infectious Diseases, Department of Internal Medicine, University of Cincinnati College of Medicine, Cincinnati, Ohio, United States of America; 3 Department of Epidemiology, Rollins School of Public Health, Emory University, Atlanta, Georgia, United States of America; 4 Cincinnati Veterans Affairs Medical Center, Cincinnati, Ohio, United States of America; Azienda Ospedaliero Universitaria Careggi, ITALY

## Abstract

**Background and objectives:**

Research on Legionnaires’ Disease (LD) suggests there may be long-term health complications, but data are limited. This study investigated whether Intensive Care Unit (ICU) admission during LD hospitalization may be associated with adverse health outcomes and characterized subsequent discharge diagnoses in patients with LD up to 5 years post-LD.

**Methods:**

We conducted a retrospective case series study with follow up for 5 years among patients hospitalized at a Department of Veterans Affairs (VA) Medical Center between 2005 and 2010 with LD. Data were collected from medical records on health history, LD severity (including ICU admission), and discharge diagnoses for 5 years post-LD or until death. We used ordinal logistic regression to explore associations between ICU admission and hospitalizations post-LD. Frequency counts were used to determine the most prevalent discharge diagnoses in the 5 years post-LD.

**Results:**

For the 292 patients with laboratory-confirmed LD, those admitted to the ICU during LD hospitalization were more likely to have a greater number of hospitalizations within 5 years compared to non-ICU patients (OR_Hosp_ 1.92 CI_95%_ 1.25, 2.95). Fifty-five percent (161/292) had ≥ 1 hospitalization within 5 years post-LD. After accounting for pre-existing diagnosis codes in patients with at least one hospitalization in the 2 years prior to LD (n = 77/161 patients, 47.8%), three of the four most frequent new diagnoses in the 5 years post-LD were non-chronic conditions: acute renal failure (n = 22, 28.6%), acute respiratory failure (n = 17, 22.1%) and unspecified pneumonia (n = 15, 19.5%).

**Conclusions:**

Our findings indicate that LD requiring ICU admission is associated with more subsequent hospitalizations, a factor that could contribute to poorer future health for people with severe LD. In addition to chronic conditions prevalent in this study population, we found new diagnoses in the 5-year post-LD period including acute renal failure. With LD incidence increasing, more research is needed to understand conditions and factors that influence long term health after LD.

## Introduction

Legionnaires’ Disease (LD) is a pneumonia with potential multi-system sequelae caused by bacteria in the genus *Legionella* [1]. The LD incidence in the United States (U.S.) has been increasing for years [2, 3], with almost 10,000 cases reported to the U.S. Centers for Disease Control and Prevention in 2018 [4]. Because it is estimated that fewer than 5% of LD cases are identified [1], the potential burden of *Legionella* disease is likely much larger than reported. This under-recognition of LD cases complicates the examination of long-term health outcomes associated with LD, an important area of study given the adverse outcomes across organ systems from *Legionella* infection [5–8] and the effect on quality of life [9, 10].

While reports have assessed acute outcomes of LD [11, 12], research on long term health effects is limited, with most studies following LD cases for less than two years. In a 2014 cross-sectional study of health outcomes after Q-fever and LD, the 190 LD patients more frequently reported fatigue and poorer quality of life one year after their infection [13]. Another study of 122 individuals who contracted LD during an outbreak found that 64% had not attained their pre-illness quality of life 2 years following LD [14]. Lung imaging of 86 individuals from that same LD outbreak found that 24% had pulmonary abnormalities in follow-up one year or later after acute infection [15]. A 2010 study comparing LD caused by *L*. *pneumophila* and *L*. *longbeachae* found a total of four re-admissions within one year after discharge among 20 patients with LD caused by the former pathogen compared to two re-admissions in 24 patients with LD caused by the latter [16]. While these studies followed LD cases for longer durations than most, no studies to our knowledge have followed subjects beyond 2 years after LD diagnosis, and none have explored whether LD requiring ICU admission is associated with worse long-term health outcomes compared to non-ICU care for LD.

To address knowledge gaps in understanding the health consequences of LD, our study leveraged the national electronic health record (EHR) system [17, 18] of the Veterans Health Administration (VHA) in the U.S. Department of Veterans Affairs (VA) to 1) investigate whether LD requiring ICU admission is associated with a greater likelihood of adverse health outcomes than non-ICU-admitted cases, and 2) explore the frequency of and reasons for subsequent hospitalizations up to 5 years post-LD. Our findings reinforce the potential for LD to have serious outcomes in the months and years after *Legionella* infection.

## Materials and methods

### Study population

The VHA healthcare system is the largest integrated healthcare system in the U.S. and provides care to nearly 9 million enrolled military veterans at 170 medical centers across the U.S. and territories. Included were individuals discharged from a VA acute care facility from federal fiscal year (FY) 2005 through FY 2010 (October 1, 2004 –September 30, 2010) with an International Classification of Diseases, 9^th^ revision, Clinical Modification, (ICD-9-CM) diagnosis code for LD (482.84).

The study protocol was reviewed by the Cincinnati VA Medical Center Research Service for adherence to VA patient privacy policy and approved by the University of Cincinnati Institutional Review Board (IRB), the IRB of record for the Cincinnati VA Medical Center (IRB #2017–3328). The IRB waived informed consent for this retrospective case series study. No patient medical charts were altered in the course of this study, and the rights and welfare of patients included in the study were not adversely affected. Identifiable information was used for chart abstractions, and use of patient record data for analysis was approved by the VA Data Access Request Tracker.

### Data collection

To identify patients with an LD discharge diagnosis between FY 2005 and FY 2010, we queried the VHA Patient Treatment Files (PTF) for the ICD-9-CM code for LD. For patients identified as having LD via this initial search, we confirmed the LD diagnosis through medical chart review for evidence of laboratory verification via urine antigen test, respiratory culture, or serology.

For all patients with laboratory-confirmed LD (“qualifying LD admission”), a clinician (N.R.) abstracted the EHR for details on clinical course during the admission for: pertinent respiratory and immune history, including chronic obstructive pulmonary disease (COPD), emphysema, asthma, cancer, smoking and human immunodeficiency virus (HIV) status; intensive care unit (ICU) admission data, including mechanical ventilation and sepsis diagnoses during the qualifying LD admission; and radiologic imaging results. ICU admission was considered a surrogate of LD case severity [5, 10].

To collect data on long term health outcomes, we searched LD case patients’ medical records in the PTF for all subsequent inpatient hospitalizations at any VA hospital within the 5-year period after the LD qualifying admission. We recorded all ICD-9-CM discharge codes associated with any VA hospitalization during this follow-up period to investigate associations with LD case severity.

### Statistical analysis

To assess differences in demographic characteristics (age, gender, race/ethnicity), pre-existing conditions (COPD, asthma, emphysema, HIV, cancer), indicators of disease severity (mechanical ventilation, diagnosis of sepsis), and length of stay for the qualifying LD admission between ICU-admitted and non-ICU-admitted patients, we used two-sided t-tests for continuous variables and Fisher’s exact tests for categorical variables.

We evaluated several criteria to determine adverse health outcomes in ICU and non-ICU groups. We used ordinal logistic regression and unconditional logistic regression to assess the association between ICU admission and number of hospitalizations in the 5-year follow-up period. For ordinal regression analyses, we defined the outcome variable by three groups: 0–1 subsequent hospitalizations, 2 subsequent hospitalizations, and 3 or more subsequent hospitalizations, according to the sample’s hospitalization tertiles. We assessed the proportional odds assumption using log-log survival curves and goodness of fit tests. To assess confounding, we condensed any diagnosis of emphysema, asthma, and COPD into one dichotomous chronic respiratory history variable to avoid over stratification of our limited sample. Similarly, we also condensed any diagnosis of HIV and current or past history of cancer into one dichotomous variable representing immunocompromised status. In the fully adjusted models, we included variables for age, respiratory history, smoking history, immunocompromised status, and race. To test for interaction, we jointly tested ICU with chronic respiratory history, age, and immunocompromised status, followed by backwards elimination for confounding assessment. We also used a separate model using only respiratory history as a confounder to assess the odds ratio for subsequent hospitalizations in ICU versus non-ICU LD patients.

To evaluate differences in time to first hospitalization subsequent to the qualifying LD admission, between ICU and non-ICU groups, we fit Cox proportional hazard models to calculate hazard ratios. We considered age, respiratory history, smoking, and immunocompromised status as confounders in these models. A log-rank test was used to test the equality of the survival curves for ICU and non-ICU LD patients.

We used frequency counts to identify the primary ICD-9-CM codes most often observed at the first subsequent hospitalization after the qualifying LD admission; we used the Fisher’s exact test to assess differences in frequencies of codes at subsequent hospitalizations between ICU and non-ICU LD cases. We also used frequency counts to find the primary ICD-9-CM codes most often newly observed in patients hospitalized in the 5 FYs after the qualifying LD admission. Patients were included in this analysis if they had at least one VA hospitalization in the two FYs prior to the qualifying LD admission (i.e., the pre-LD period) and at least one VA hospitalization in the 5 FYs after the qualifying LD admission; if diagnosis codes were present in a patient’s medical record pre-LD, those codes were not included in the frequency count for the post-LD period. Diagnosis codes in the qualifying LD admission were not used for this adjustment. Remaining diagnosis codes in the post-LD period were counted only once per patient at first appearance in the medical record. Frequencies for post-LD diagnosis codes were calculated at six time points: 6 months post-LD and annually thereafter for 5 FYs.

## Results

**Fig 1:**
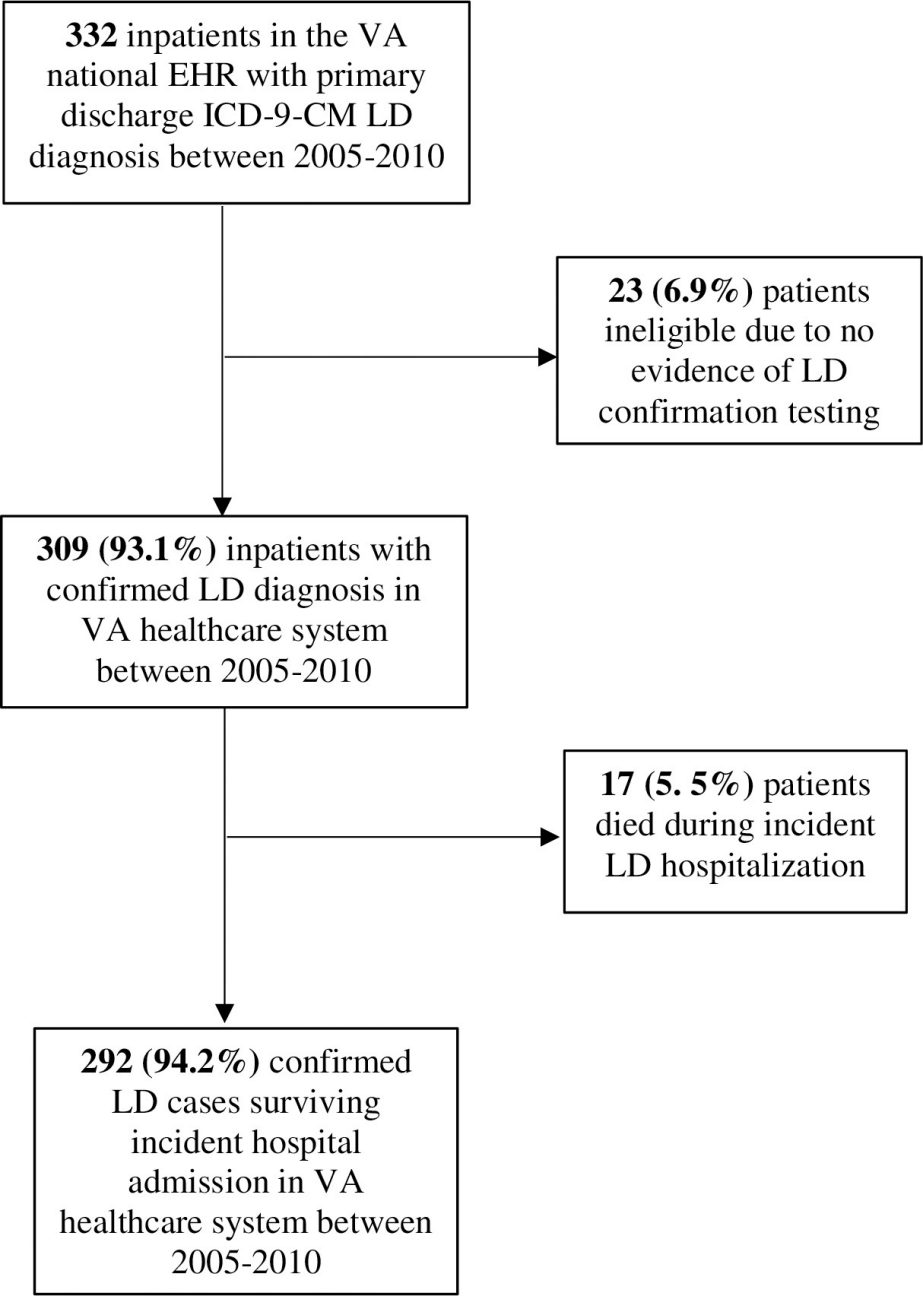
Study population selection from 332 VA inpatients discharged with primary ICD-9-CM diagnosis Legionnaires' Disease (LD) between 2005–2010. Abbreviations: VA U.S. Department of Veterans Affairs; LD Legionnaires’ Disease; ICD-9-CM International Classification of Diseases, 9th revision, Clinical Modification; EHR electronic health record.

The initial search of the national VA EHR for the ICD-9-CM diagnosis code for LD yielded 332 possible cases between FY 2005 –FY 2010 (Fig 1). Twenty-three patients (6.9%) were excluded due to no evidence of LD laboratory testing in their medical record. Seventeen patients (5.5%) died during their LD admission. While these individuals were excluded from the final sample, demographic and select medical data can be found in S1 Table. Our final cohort included 292 LD cases for analyses of long term health outcomes.

The median age of study subjects was 61 years, and the cohort was primarily male (97.3%), white (73.3%), and a reported smoker (65.8%) (Table 1). While the proportions of patients presenting with asthma, emphysema, cancer and HIV at their qualifying LD admission were comparable between ICU-admitted patients and non-ICU-admitted patients, the prevalence of COPD was significantly higher among the ICU group than the non-ICU group (36.2% vs. 21.0%; p < 0.01). Compared to the non-ICU group, ICU patients were also more frequently diagnosed with sepsis during admission (31.5% vs. 4.9%; p < 0.01)and had a longer length of stay (16.8 days vs. 6.1 days; p < 0.01). Thirty-six (27.7%) of the patients with ICU admission were intubated. Survival curves between the ICU and non-ICU groups were not significantly different (p = 0.87).

**Table 1:** Demographics, select medical history, Legionnaires' disease severity indicators, and mortality of 292 inpatients surviving a hospitalization with laboratory-confirmed Legionnaires’ disease between 2005–2010 in any U.S. VA medical facility.

Category	All patients (n = 292)	ICU admitted patients (n = 130)	Non-ICU admitted patients (n = 162)	*p*^*a*^
**Demographic data**				
Age (median, IQR)	61, 56–71	61, 58–71	61.5, 56–71	0.72
Men, n (%)	284 (97.3)	125 (96.1)	159 (98.1)	0.47
Women, n (%)	8 (2.7)	5 (3.9)	3 (1.9)	
White, n (%)	214 (73.3)	96 (73.9)	118 (72.8)	0.73
Black, n (%)	69 (23.6)	31 (23.9)	38 (23.5)	
Hispanic, n (%)	2 (0.7)	0 (0)	2 (1.2)	
Asian, n (%)	2 (0.7)	1 (0.8)	1(0.6)	
Native American, n (%)	5 (1.7)	2 (1.5)	3 (1.9)	
**Pre-existing conditions, n (%)**				
Smoker at incident admission	192 (65.8)	89 (68.5)	103 (63.6)	0.39
HIV positive	10 (3.4)	4 (3.1)	6 (5.5)	0.77
COPD	81 (27.7)	47 (36.2)	34 (21)	**<0.01**
Emphysema	2 (0.7)	1 (0.8)	1 (0.1)	0.88
Asthma	11 (3.8)	5 (3.9)	6 (3.7)	0.95
Cancer	66 (22.6)	24 (18.5)	42 (25.9)	0.13
**Indicators of disease severity**				
Diagnosed septic at admission, n (%)	49 (16.8)	41(31.5)	8 (4.9)	**<0.01**
Length of stay, days (sd)	11.5 (±19.45)	16.8 (± 34.5)	6.1 (± 4.4)	**<0.01**
**Mortality in follow-up, n (%)**	90 (30.8)	41 (31.5)	49 (30.3)	0.81

Abbreviations: VA Department of Veterans Affairs, ICU Intensive Care Unit, HIV Human Immunodeficiency Virus, COPD Chronic Obstructive Pulmonary Disease, IQR Interquartile Range, sd Standard Deviation

^a^p-values assessed with t-test of differences in means for continuous variables and Fisher's Exact Test for dichotomous variables

Of the 292 patients in the sample, 131 (45%) had no subsequent hospitalization recorded in the VA EHR in the 5 years following their qualifying LD admission. Thirty-four (26%) of these 131 individuals died within the follow-up period. For the remaining 161 patients with record of at least one subsequent hospitalization in the following 5 years, 56 (35%) died in the follow-up period. The 30-day all-cause readmission rate from the LD qualifying admission was 16.2% (26/161).

Average time to first hospitalization after the qualifying LD admission was 29.5 months in ICU-admitted LD patients and 36.1 months in non-ICU-admitted LD patients (HR_Crude_ 1.46, 95% CI_:_ 1.06, 2.02 p = 0.02). While this association was attenuated after adjustment for respiratory history alone (HR_RespAj_ 1.37, 95% CI: 0.98, 1.90 p = 0.06), it remained significant upon adjustment for age, smoking, respiratory history, and immunocompromised status (HR_Adj_ 1.46 CI_95%Adj_ 1.05, 2.04 p = 0.02). Age, respiratory history, smoking, immunocompromised status, and ICU admission were assessed for proportional hazards, and found to have no significant violations in graphical log-log survival curves, goodness of fit testing, and time-dependent variable testing.

The most common discharge diagnosis code for the first subsequent hospitalization was unspecified pneumonia (n = 14, 8.7%) (Table 2). This proportion was substantially more than the next diagnosis of coronary atherosclerosis of coronary artery disease (n = 6, 3.7%), and remained so even after exclusion of potential readmission hospitalizations occurring within 30 (n = 26) and 60 (n = 43) days of the qualifying LD admission (S2 and S3 Tables). While more diagnoses of respiratory illness including pneumonia were observed at first hospitalization post-LD among ICU cases (n = 15) than non-ICU cases (n = 10), the difference was not statistically significant and was attenuated further upon adjustment for the aforementioned pre-existing conditions (Table 3).

**Table 2:** Frequency^a^ of primary ICD-9-CM discharge diagnosis code of first subsequent hospitalization after incident Legionnaires' disease hospitalization among patients to any U.S. VA medical facility, 2005–2010.

Diagnosis (ICD-9-CM Code)	Frequency (n = 161^b^)	Percentage
Pneumonia, unspecified (486.0)	14	8.7
Coronary Atherosclerosis of Coronary Artery (414.10)	6	3.7
Obstructive Chronic Bronchitis with Acute Exacerbation (491.21)	4	2.5
Depressive Disorder (311)	4	2.5
Hyperpotassemia (276.7)	3	1.9
Acute Pancreatitis (577.0)	3	1.9
Urinary Tract Infection (599.0)	3	1.9
Cellulitis of Leg (682.6)	3	1.9
Drug Induced Mood Disorder (292.84)	3	1.9
Pulmonary Embolism and Infarction (415.19)	3	1.9
Cerebral Artery Occlusion with Cerebral Infarction (434.91)	3	1.9
Legionnaires' Disease (482.84)	3	1.9
Acute Respiratory Failure (518.81)	3	1.9
Chest Pain (786.59)	3	1.9
Malignant Neoplasm of Bronchus/Lung (162.9)	2	1.2
Secondary Malignant Neoplasm of Brain/Spine (198.3)	2	1.2
Secondary Malignant Neoplasm of Bone (198.5)	2	1.2
Acute Kidney Failure (584.9)	2	1.2
Syncope and Collapse (780.2)	2	1.2
Bipolar Disorder (296.80)	2	1.2
Non-ST-Elevation Myocardial Infarction (410.71)	2	1.2
Atrial Fibrillation (427.31)	2	1.2
Post-operation Infection (998.59)	2	1.2

Abbreviation: VA, Department of Veterans Affairs

^a^ Table lists the diagnoses for which the frequency was ≥ 2. There were 70 additional diagnoses with a frequency of 1 (See S4 Table).

^b^ Number of patients in the study who had at least one VA hospitalization after the qualifying Legionnaires’ disease admission.

**Table 3:** Crude and adjusted odds ratios for subsequent hospitalizations between ICU versus non-ICU admitted veterans hospitalized for Legionnaires’ disease in any U.S. VA medical facility, 2005–2010.

	OR (95%CI)	*p*	aOR^c^ (95% CI)	*p*
Hospitalizations after incident LD admission in 5-year follow-up^a^	1.92 (1.25, 2.95)	**<0.01**	1.93 (1.25, 3.00)	**<0.01**
First hospitalization for respiratory diagnosis after incident LD admission^b^^,^^d^	1.98 (0.86, 4.57)	0.11	1.77 (0.75, 4.18)	0.19
First hospitalization for pneumonia diagnosis after incident LD admission^b^	2.05 (0.77, 5.44)	0.15	1.74 (0.63, 4.80)	0.28

Abbreviations: aOR, adjusted odds ratio; CI, confidence interval; ICU Intensive Care Unit; LD, Legionnaires' Disease; OR, odds ratio; VA, Department of Veterans Affairs

^a^Obtained using ordinal logistic regression

^b^Obtained using logistic regression

^c^Adjusted for age, respiratory history, immunocomprised history, and smoking at incident LD admission.

^d^Respiratory diagnoses included LD, unspecified pneumonia, acute bronchitis, *Klebsiealla* pneumonia, pleural effusion, pneumothorax, obstructive chronic bronchitis with acute exacerbation, acute respiratory failure and tachypnea.

Cases admitted to the ICU during the qualifying admission were more likely to have a greater number of hospitalizations within 5 years compared to non-ICU cases (OR_Hosp_ 1.92 CI_95%_ 1.25, 2.95 p<0.01) (Table 3). This association remained significant after adjusting for age, immunocompromised status, smoking, and respiratory history (OR_HospAdj_ 1.93 CI_95%Adj_ 1.25, 3.00 p<0.01).

To assess new health conditions after LD, we reviewed ICD-9-CM coding for hospitalizations in the 5 years post-LD after first removing codes that appeared in the patients’ medical records in the 2 years prior to LD. Of the 161 patients with at least one VA hospitalization in the 5 years after LD, 77 (48%) also had at least one VA hospitalization in the 2 years pre-LD. The most common new discharge diagnosis in the entire 5-year follow-up period for subsequent admissions in the 77 patients was acute renal failure (n = 22, 28.6%) (Table 4). When the most frequent new discharge diagnoses were assessed in cumulative incremental time periods at 6 months and annually thereafter for 5 years, several diagnoses were present as a frequent diagnosis in all periods (Table 4). Most of the frequently observed discharge diagnoses found in LD patients during all post-LD time periods in the 5-year follow-up were common chronic diagnoses such as hypertensive chronic kidney disease (n = 20, 26%) and atrial fibrillation (n = 13, 16.9%), as were the frequent diagnoses in individual time periods that were not present in all time periods (S5 Table). However, also frequently included in all time periods were acute diagnoses such as the aforementioned acute renal failure, acute respiratory failure (n = 17, 22.1% after 5 years) and unspecified pneumonia (n = 15, 19.5% after 5 years). Stratification of the frequencies of renal diseases in Table 4 [acute renal failure (584.9), hypertensive chronic kidney disease (403.90), and chronic kidney disease (585.9)] by ICU versus non-ICU admission during the LD qualifying visit was unremarkable, likely a result of the low numbers of cases in each category for each time period (S6 Table).

**Table 4:** Most frequent^a^ administrative discharge diagnosis codes after diagnosis of Legionnaires’ disease, at 6 months after LD and cumulative annually for five Federal Fiscal Years^b^ (n = 77)–frequent diagnoses that were present in all six time increments.

Diagnosis (ICD-9-CM Code)	Frequency^c^ (percent of patients)					
	6 months (n = 34)	1 year (n = 45)	2 years (n = 58)	3 years (n = 62)	4 years (n = 73)	5 years (n = 77)
Acute renal failure, unspecified (584.9)	8 (23.5)	9 (20)	10 (17.2)	14 (22.6)	18 (24.7)	22 (28.6)
Acute respiratory failure (518.81)	7 (20.6)	7 (15.6)	9 (15.5)	13 (21.0)	15 (20.5)	17 (22.1)
Pneumonia, organism unspecified (486)	7 (20.6)	7 (15.6)	10 (17.2)	10 (16.1)	12 (16.4)	15 (19.5)
Hypertensive chronic kidney disease, unspecified (403.90)	6 (17.6)	9 (20)	13 (22.4)	14 (22.6)	17 (23.3)	20 (26.0)
*Clostridium difficile* (008.45)	6 (17.6)	6 (13.3)	9 (15.5)	9 (14.5)	9 (12.3)	9 (11.7)
Atrial fibrillation (427.31)	5 (14.7)	6 (13.3)	9 (15.5)	9 (14.5)	12 (16.4)	13 (16.9)
Anemia, unspecified (285.9)	5 (14.7)	7 (15.6)	7 (12.1)	8 (12.9)	9 (12.3)	9 (11.7)
Chronic kidney disease, unspecified (585.9)	4 (11.8)	6 (13.3)	11 (19.0)	12 (19.4)	15 (20.5)	15 (19.5)
Hyperpotassemia (276.7)	4 (11.8)	5 (11.1)	5 (8.6)	8 (12.9)	8 (11.0)	8 (10.4)
Congestive heart failure, unspecified (428.0)	4 (11.8)	6 (13.3)	7 (12.1)	8 (12.9)	13 (17.8)	7 (12.1)
Obstructive sleep apnea (327.23)	3 (8.8)	4 (8.9)	5 (8.6)	7 (11.3)	9 (12.3)	12 (15.6)
Hypertension disease, unspecified (401.9)	3 (8.8)	4 (8.9)	7 (12.1)	8 (12.9)	10 (13.7)	11 (14.3)

Abbreviation: ICD-9-CM, International Classification of Diseases, 9^th^ Revision, Clinical Modification; LD, Legionnaires’ disease

^a^ For each patient, ICD-9-CM discharge diagnosis codes that were present in the 2 years prior to LD were not used for frequency calculations in the post-LD period. Each code remaining in the post-LD period was counted only once for each patient at first appearance in the medical record. The most frequent ICD-9-CM codes present in all six time period and for which about 10% of the cohort had the code are presented.

^b^ In the United States, the Federal Fiscal Year is October 1 to September 30.

^c^ The number of patients in each cumulative time period is based on the number of patients who survived to that time period and who had a hospitalization in the VA system up to the time period.

Discharge coding for the 22 patients with acute renal failure post-LD but not pre-LD was examined further to see if these patients had other kidney-related conditions diagnosed in the pre-LD period. Of the 22 patients, 17 (77.3%) had no codes in the 2-years pre-LD corresponding with nephritis, nephrotic syndrome, and nephrosis (codes 580–589) or with hypertensive chronic kidney disease (codes 403 and 404). Eight of the 22 patients (36.4%) had no other kidney-related discharge diagnosis codes in the 5-years post LD. We also examined coding for the 22 patients in the qualifying LD admission for acute kidney failure; thirteen (59%) did not have the code in that visit. Finally, we reviewed the pre-LD period for the 22 patients for diabetes mellitus (code 250.*) since this condition can impact renal disease; 9 of the patients did not have diabetes mellitus codes in the pre-LD period.

## Discussion

As the first large study to explore health-related outcomes of LD patients beyond 2 years, this work offers insights into the longer-term health outcomes potentially associated with LD. The 30-day all-cause readmission rate of 16.2% is similar to published 30-day readmission rates for pneumonia [19]. This substantiates that outcomes from this study may be comparable to outcomes for patients in other healthcare systems.

Unspecified pneumonia was the most frequent discharge diagnosis for a first hospitalization after an LD admission, occurring more than twice as frequently as the next most common diagnosis. When we excluded discharge diagnosis codes from first hospitalizations within 30 or 60 days of the LD admission, unspecified pneumonia remained the most frequent diagnosis, indicating that these pneumonias were likely not continuations of the incident LD episode.

With so little known about the long-term health consequences of LD, especially among cases not necessarily linked to an LD outbreak, this large descriptive study generates numerous hypotheses worthy of further exploration. Our study offers insight as to whether LD resulting in ICU admission impacts long term health. In our analysis, LD requiring ICU admission was associated with a higher mean number of hospitalizations in the 5-year follow-up period and a shorter time to first subsequent hospitalization than non-ICU LD cases. This included a non-significant but increased likelihood of subsequent hospitalizations for pneumonia. Our findings suggest that LD resulting in an ICU admission may play a role in increased morbidity after the LD episode. Future studies could attempt to discern if these findings are unique to LD, or if similar outcomes are observed following hospitalizations for pneumonias of other etiologies.

Intensive care unit admission can be considered a surrogate for severity of illness, and the patients in our study with ICU admission during LD had significantly more severe illness than those not admitted to the ICU. We excluded the 17 patients who died with LD during their stay since they could not be assessed for long term health outcomes; all 17 patients were admitted to the ICU during their LD visit and their exclusion likely attenuated some of the differences observed in disease severity in our comparison of ICU- and non-ICU patients. Admission to the ICU for any reason is known to be associated with subsequent morbidity, especially in elderly populations, and further research is needed to determine whether the increased risk for subsequent hospitalizations observed here is different from the risk for rehospitalization following ICU admission for other reasons [20]. But, one third of the patients in our study died within 5 years regardless of ICU admission during the LD visit, a result that suggests that hospitalization for LD itself may be an indicator of poorer long term outcomes [21]. Additional research into the outcomes of severe LD compared to less severe LD would be well served by including comparisons to ambulatory or non-hospitalized cases. Others have found that patients with pneumonia requiring hospitalization have frequent rehospitalizations and higher mortality compared to non-hospitalized patients [22, 23]; comparing LD cases to patients diagnosed with pneumonia of other etiologies will be needed to determine whether LD has a different morbidity or mortality profile over time compared to other pneumonias.

Case studies of severe *Legionella* pneumonia have reported concomitant kidney injury that range from mild, temporary impairment to damage necessitating dialysis, indicating the potential for long-term adverse renal effects secondary to infection [24–29]. In this study starting with hundreds of cases over several years, we found that chronic and acute kidney disorders were among the more frequently observed diagnoses in follow-up, with acute renal failure the most common discharge diagnosis code after 5 years of follow up. Our findings lend further support to the observation that renal disease associates with LD, both in the immediate period after LD or after a prolonged time, and is not rare. Whether this represents a true increase in long term kidney illness due to LD as opposed to other influences [30, 31] remains to be further elucidated.

Our study has some important strengths and limitations. First, this is the first study to attempt to examine health outcomes of LD cases regardless of their connection to an identified outbreak. The VA has had long-standing policy for preventing LD, including policy initiated in 2008 encouraging routine testing of pneumonia patients for LD [32]. More recent data confirm extensive LD testing in VHA [33], and the policy in effect at the timeframe of this study population likely promoted the diagnosis and therefore availability of a sizable number of LD cases for this analysis. By not being limited to outbreak-associated cases and by using medical records to examine case histories, this study avoided recall bias associated with patient self-report or recording of health metrics before and after a recognized LD outbreak [34].

Second, a particular strength of this study was the availability of medical records in the VA EHR system, allowing for long-term follow-up of a large cohort of LD patients. A comparable dataset in the U.S. is not as readily available from private sector healthcare records. The intent of this study was to identify and characterize hospitalized cases of LD in the VA medical system from FY 2005 to FY 2010, so we did not identify a comparison group, nor did we explore outpatient records related to non-hospitalized cases. Future use of the VA EHR for these efforts will build on the findings in this study.

Utilization of data in VA medical records has some limitations. First, we were reliant on administrative discharge diagnosis coding for patients that had at least one admission to a VA facility in the 2 years prior to the qualifying LD visit as an indication of pre-existing conditions and subsequent new diagnoses post-LD. Use of discharge coding for health research in VA patients is well-established [31, 35–37], including for examination of health conditions in a follow-up period [38], but coding accuracy can be an issue [39, 40]. Since we did not collect complete health histories on each case, it is possible that the conditions observed for some patients in follow-up hospitalizations were a reflection of un-coded pre-existing conditions and not new diagnoses after LD. Nonetheless, for the most frequently observed new diagnosis of acute renal failure in the 5 years after LD, most patients still had that code when a grouping of renal diagnosis codes was alternatively searched (as opposed to the exact code match) in the 2-year pre-LD period to identify a previous renal condition. Another limitation is that about half the patients with post-LD hospitalizations did not have pre-LD admissions in the VA medical record and could not be included in the analysis to assess new diagnoses post-LD. This may mean that healthier people who did not require hospitalization were excluded from our long-term analyses, biasing our findings to post-LD conditions observed in people with poorer overall health status. Even if our sample included mostly sicker patients, our conclusions would suggest that, for patients with poor health, diagnosis with LD can lead to certain conditions not present prior to LD. Furthermore, for all patients in the study, a severe LD infection resulting in an ICU stay may increase their risk of hospitalization in the following years. For these patients especially, our findings emphasize the importance of definitive diagnostic testing and expedient delivery of appropriate antibiotics after identifying pneumonia. Finally, while using VA EHR records afforded us the ability to track patients longitudinally regardless of which VA facility they visited, patients served by the VA health system are not representative of the general U.S. population; they are disproportionately male, more sick and poorer [41]. They may also receive care outside the VA system, and we did not attempt to obtain data from external healthcare providers to supplement our analyses. Despite these challenges, when adjusted for indicators of poor health status (e.g., respiratory history and immunocompromised status), the trend of increased hospitalizations after more severe LD remained significant.

## Conclusions

This retrospective case series offers unique and important insights into the long-term health of patients with LD, including the potential for the *Legionella* infection to accelerate the trajectory of other conditions such as acute renal illness. Differences between ICU-admitted and non-ICU admitted patients with LD were also highlighted, suggesting the need to follow ICU-admitted LD patients more closely after discharge to prevent readmissions and adverse health effects. Further research regarding the prevalence of pre-existing conditions and their effect on hospital course for LD and other pneumonias, differences in health outcomes after pneumonias of different etiologies, and the associations between those outcomes and the severity of incident pneumonia will add to understanding the epidemiology and health consequences of LD.

## Supporting information

S1 Table Demographics, select medical history, and Legionnaires' Disease (LD) severity indicators for 17 patients who died during qualifying LD admission, U.S. VA medical facilities, 2005–2010. (DOCX)

S2 Table Frequency of primary ICD-9-CM diagnosis codes for first subsequent hospitalization after incident LD hospitalization among patients at any US VA medical facility, 2005–2010, excluding hospitalizations within 30 days of incident LD hospitalization discharge. (DOCX)

S3 Table Frequency of primary ICD-9-CM diagnosis codes for first subsequent hospitalization after incident LD hospitalization among VA patients to any US VA medical facility, 2005–2010, excluding hospitalizations within 60 days of incident LD hospitalization discharge. (DOCX)

S4 Table Primary ICD-9-CM discharge diagnosis codes with a frequency of 1^a^ for first subsequent hospitalization after incident Legionnaires' disease hospitalization among patients to any U.S. VA medical facility, 2005–2010. (DOCX)

S5 Table Most frequent^a^ administrative discharge diagnosis codes after a diagnosis of Legionnaires’ disease, at 6 months after LD and cumulative annually for five Federal Fiscal Years^b^ (n = 77)–frequent diagnoses that were not present in all six time increments. (DOCX)

S6 Table Frequency of renal disease ICD-9-CM discharge diagnosis codes in Legionnaires’ Disease (LD) patients at 6 months after LD and cumulative annually for five Federal Fiscal Years^a^, stratified by Intensive Care Unit (ICU) versus non-ICU admission during the LD hospitalization. (DOCX)
