# Supplementary material for: Health after Legionnaires' disease: A description of hospitalizations up to 5 years after Legionella pneumonia
Source: PLoS One. 2021 Jan 11;16(1):e0245262. doi: 10.1371/journal.pone.0245262 (PMC7799844; doi:10.1371/journal.pone.0245262)
Supplement: S1 Table — (DOCX) [file pone.0245262.s001.docx]

S1 Table. Demographics, select medical history, and Legionnaires' disease (LD) severity indicators for 17 patients who died during qualifying LD admission, U.S. VA medical facilities, 2005 – 2010

| **Criterion** |  |
| --- | --- |
|  | **n (%)** |
| LD Confirmation Testing | 17 (100) |
| Men | 17 (100) |
| White | 17 (100) |
| Smoker at Incident Admission | 8 (47.1) |
| HIV Positive | 0 (0) |
| COPD | 4 (23.5) |
| Emphysema | 1 (5.9) |
| Asthma | 0 (0) |
| Cancer | 5 (29.4) |
| Diagnosed Septic at Admission | 8 (47.1) |
| Intubated during Admission | 15 (88.2) |
| ICU Admission | 17 (100) |
|  | **Time (**+**sd)** |
| Age at Admission, in years | 64.6 (+15.8) |
| Length of Stay, in days | 10.5 (+22.6) |
| Abbreviations: COPD, Chronic Obstructive Pulmonary Disease; HIV, Human Immunodeficiency Virus; ICU, Intensive Care Unit; LD, Legionnaires' Disease; sd, Standard Deviation; VA, Department of Veterans Affairs; | |
|  |  |
