# Supplementary material for: Health after Legionnaires' disease: A description of hospitalizations up to 5 years after Legionella pneumonia
Source: PLoS One. 2021 Jan 11;16(1):e0245262. doi: 10.1371/journal.pone.0245262 (PMC7799844; doi:10.1371/journal.pone.0245262)
Supplement: S3 Table — (DOCX) [file pone.0245262.s003.docx]

**Supporting Information**

S3 Table. Frequency of primary ICD-9-CM diagnosis codes for first subsequent hospitalization after incident LD hospitalization among VA patients to any US VA medical facility, 2005 – 2010, excluding hospitalizations within 60 days of incident LD hospitalization discharge

| **Diagnosis** | **ICD-9-CM Code** | **Frequency (n=118)** | **Percentage** |
| --- | --- | --- | --- |
| Pneumonia, unspecified | 486 | 11 | 9.3 |
| Coronary Atherosclerosis of Coronary Artery | 414.01 | 6 | 5.1 |
| Obstructive Chronic Bronchitis with Acute Exacerbation | 491.21 | 4 | 3.4 |
| Depressive Disorder | 311 | 4 | 2.5 |
| Acute Pancreatitis | 577.0 | 3 | 2.5 |
| Cellulitis of Leg | 682.6 | 3 | 2.5 |
| Drug Induced Mood Disorder | 292.84 | 3 | 2.5 |
| Acute Respiratory Failure | 518.81 | 3 | 2.5 |
| Congestive Heart Failure, Unspecified | 428.0 | 3 | 2.5 |
| Abbreviations: LD, Legionnaires’ disease; ICD-9-CM  International Classification of Diseases, 9th revision, Clinical Modification; VA Department of Veterans Affairs | | | |
|  |  |  |  |
