# Supplementary material for: Health after Legionnaires' disease: A description of hospitalizations up to 5 years after Legionella pneumonia
Source: PLoS One. 2021 Jan 11;16(1):e0245262. doi: 10.1371/journal.pone.0245262 (PMC7799844; doi:10.1371/journal.pone.0245262)
Supplement: S4 Table — (DOCX) [file pone.0245262.s004.docx]

S4 Table. Primary ICD-9-CM discharge diagnosis codes with a frequency of 1^a^ for first subsequent hospitalization after incident Legionnaires' disease hospitalization among patients to any U.S. VA medical facility, 2005 – 2010.

| **ICD-9-CM Code** | **Description** | |  |
| --- | --- | --- | --- |
| 566 | Rectal Abscess | |  |
| 110.4 | Dermatophytosis of Foot | | |
| 110.5 | Dermatophytosis of Body | | |
| 153.4 | Malignant Neoplasm Cecum | | |
| 157.9 | Vitreous Operation | |  |
| 188.2 | Malignant Neoplasm of Bladder | | |
| 188.8 | Malignant Neoplasm of Bladder | | |
| 188.9 | Malignant Neoplasm of Bladder | | |
| 211.3 | Benign Neoplasm of Colon | | |
| 285.9 | Anemia, unspecified | |  |
| 287.5 | Thrombocytopenia, unspecified | | |
| 413.9 | Angina pectoris | |  |
| 414.8 | Chronic Ischemic Heart Disease | | |
| 416.0 | Primary Pulmonary Hypertension | | |
| 428.0 | Congestive Heart Failure, unspecified | | |
| 458.9 | Hypotension | |  |
| 466.0 | Acute Bronchitis | |  |
| 482.0 | Pneumonia due to Klebsiella Pneumoniae | | |
| 511.8 | Pleural Effusion | |  |
| 512.8 | Pneumothorax | |  |
| 558.9 | Noninfectious Gastroenteritis and Colitis | | |
| 560.1 | Paralytic Ileus | |  |
| 571.1 | Acute Alcoholic Hepatitis | | |
| 682.5 | Cellulitis of Buttocks | |  |
| 682.7 | Cellulitis of Foot | |  |
| 721.1 | Cervical Spondylosis with Myelopathy | | |
| 724.2 | Lumbago |  |  |
| 781.2 | Abnormal Gait | |  |
| 922.1 | Contusion of Chest Wall | | |
| 204.10 | Chronic Lymphoid Leukemia | | |
| 250.70 | Diabetes II with Peripheral Circulatory Disorders | | |
| 250.72 | Diabetes II With Peripheral Circulatory Disorders Uncontrolled | | |
| 276.51 | Dehydration | |  |
| 291.81 | Alcohol Withdrawal | |  |
| 295.70 | Schizoaffective Disorder | | |
| 296.20 | Major Depressive Affective Disorder | | |
| 296.90 | Episodic Mood Disorder | | |
| 300.01 | Panic Disorder without Agoraphobia | | |
| 303.91 | Alcohol Dependence, continuous | | |
| 304.21 | Cocaine dependence Continuous | | |
| 305.01 | Alcohol Abuse Continuous | | |
| 309.81 | Posttraumatic Stress Disorder |  |  |
| 338.18 | Acute Postoperative Pain | | |
| 398.91 | Rheumatic Heart Failure | | |
| 414.00 | Coronary Atherosclerosis of unspecified vessel type | | |
| 428.23 | Acute on Chronic Systolic Heart Failure | | |
| 428.30 | Diastolic Heart Failure | | |
| 424.11 | Aortic Vascular Disorder | | |
| 511.81 | Malignant Pleural Effusion | | |
| 535.40 | Other Specified Gastritis without Hemorrhage | | |
| 553.21 | Incisional Hernia | |  |
| 562.11 | Diverticulitis of Colon without Hemorrhage | | |
| 562.12 | Diverticulitis of Colon with Hemorrhage | | |
| 564.00 | Constipation | |  |
| 599.70 | Hematuria | |  |
| 600.01 | Hypertrophy (benign) of Prostrate with Urinary Obstruction | | |
| 707.10 | Ulcer of Lower Limb | |  |
| 707.13 | Ulcer of Ankle | |  |
| 715.35 | Osteoarthritis Pelvic region and Thigh | | |
| 721.90 | Spondylosis without myelopathy | | |
| 728.88 | Rhabdomyolysis | |  |
| 780.39 | Convulsions | |  |
| 786.06 | Tachypnea | |  |
| 787.03 | Vomiting Alone | |  |
| 788.30 | Urinary Incontinence | |  |
| 814.03 | Closed Wrist Fracture | | |
| 823.00 | Closed Fracture of Upper End Tibia | | |
| 997.62 | Infection of Amputation Stump | | |
| 997.69 | Amputation Stump Complication | | |
| 999.32 | Bloodstream Infection due to Central Venous Catheter | | |

^a^ See Table 2 of the main paper for ICD-9-CM codes with a frequency greater than 1.
