# Supplementary material for: Health after Legionnaires' disease: A description of hospitalizations up to 5 years after Legionella pneumonia
Source: PLoS One. 2021 Jan 11;16(1):e0245262. doi: 10.1371/journal.pone.0245262 (PMC7799844; doi:10.1371/journal.pone.0245262)
Supplement: S5 Table — (DOCX) [file pone.0245262.s005.docx]

S5 Table. Most frequent^a^ administrative discharge diagnosis codes after a diagnosis of Legionnaires’ disease, at 6 months after LD and cumulative annually for five Federal Fiscal Years^b^ (n=77) – frequent diagnoses that were not present in all six time increments.

| **Diagnosis (ICD-9-CM Code),**  **by cumulative increment** | **Frequency^c^ (percent of patients)** | |
| --- | --- | --- |
| **6 months (n=34)** |  |  |
| Edema (782.3) | 5 (14.7) |  |
| Dehydration (276.51) | 4 (11.8) |  |
| Anemia in chronic kidney disease (285.21) | 3 (8.8) |  |
| Polyneuropathy in diabetes (357.2) | 3 (8.8) |  |
| Subendocardial infarction (410.71) | 3 (8.8) |  |
| LD (482.84)^d^ | 3 (8.8) |  |
|  |  |  |
| **1 Year (n=45)** |  |  |
| Edema (782.3) | 5 (11.1) |  |
| Other and unspecified hyperlipidemia (272.4) | 4 (8.9) |  |
| Dehydration (276.51) | 4 (8.9) |  |
| Anemia in chronic kidney disease (285.21) | 4 (8.9) |  |
| Coronary artery disease of native coronary artery (414.01) | 4 (8.9) |  |
| LD (482.84)^d^ | 4 (8.9) |  |
| Chronic airway obstruction, not elsewhere classified (496) | 4 (8.9) |  |
|  |  |  |
| **2 Years (n=58)** |  |  |
| Coronary artery disease of native coronary artery (414.01) | 7 (12.1) |  |
| Chronic airway obstruction, not elsewhere classified (496) | 7 (12.1) |  |
| Diabetes mellitus (250.00) | 6 (10.3) |  |
| Other and unspecified hyperlipidemia (272.4) | 6 (10.3) |  |
| Hypertensive chronic kidney disease, unspecified (403.91) | 6 (10.3) |  |
| Methicillin susceptible Staphylococcus aureus (041.11) | 5 (8.6) |  |
| Depressive disorder, not elsewhere classified (311) | 5 (8.6) |  |
| Polyneuropathy in diabetes (357.2) | 5 (8.6) |  |
| Diastolic heart failure, unspecified (428.30) | 5 (8.6) |  |
| LD (482.84)^d^ | 5 (8.6) |  |
| End stage renal disease (585.6) | 5 (8.6) |  |
| Edema (782.3) | 5 (8.6) |  |
| Infection with microorganisms resistant to penicillins (V09.0) | 5 (8.6) |  |
|  |  |  |
| **3 Years (n=62)** |  |  |
| Chronic airway obstruction, not elsewhere classified (496) | 9 (14.5) |  |
| Diabetes mellitus (250.00) | 8 (12.9) |  |
| Coronary artery disease of native coronary artery (414.01) | 8 (12.9) |  |
| Polyneuropathy in diabetes (357.2) | 7 (11.3) |  |
| Diastolic heart failure, unspecified (428.30) | 7 (11.3) |  |
| Methicillin susceptible Staphylococcus aureus (041.11) | 6 (9.7) |  |
| Diabetes with neurological manifestations (250.60) | 6 (9.7) |  |
| Other and unspecified hyperlipidemia (272.4) | 6 (9.7) |  |
| Hypertensive chronic kidney disease, unspecified (403.91) | 6 (9.7) |  |
| Esophageal reflux (530.81) | 6 (9.7) |  |
| Urinary tract infection, site not specified (599.0) | 6 (9.7) |  |
| Infection with microorganisms resistant to penicillins (V09.0) | 6 (9.7) |  |
|  |  |  |
| **4 Years (n=73)** |  |  |
| Coronary artery disease of native coronary artery (414.01) | 14 (19.2) |  |
| Diabetes mellitus (250.00) | 13 (17.8) |  |
| Other and unspecified hyperlipidemia (272.4) | 11 (15.1) |  |
| Esophageal reflux (530.81) | 11 (15.1) |  |
| Chronic airway obstruction, not elsewhere classified (496) | 10 (13.7) |  |
| Polyneuropathy in diabetes (357.2) | 9 (12.3) |  |
| Diabetes with neurological manifestations (250.60) | 8 (11.0) |  |
| Diastolic heart failure, unspecified (428.30) | 8 (11.0) |  |
| Tobacco use disorder (305.1) | 7 (9.6) |  |
| Urinary tract infection, site not specified (599.0) | 7 (9.6) |  |
| Long-term (current) use of anticoagulants (V58.61) | 7 (9.6) |  |
|  |  |  |
| **5 Years (n=77)** |  |  |
| Coronary artery disease of native coronary artery (414.01) | 16 (20.8) |  |
| Esophageal reflux (530.81) | 16 (20.8) |  |
| Diabetes mellitus (250.00) | 14 (18.2) |  |
| Other and unspecified hyperlipidemia (272.4) | 12 (15.6) |  |
| Chronic airway obstruction, not elsewhere classified (496) | 11 (14.3) |  |
| Tobacco use disorder (305.1) | 10 (13.0) |  |
| Polyneuropathy in diabetes (357.2) | 10 (13.0) |  |
| Diabetes with neurological manifestations (250.60) | 9 (11.7) |  |
| Peripheral vascular disease, unspecified (443.9) | 9 (11.7) |  |
| Dehydration (276.51) | 8 (10.4) |  |
| Diastolic heart failure, unspecified (428.30) | 8 (10.4) |  |
| Long-term (current) use of anticoagulants (V58.61) | 8 (10.4) |  |

Abbreviations: ICD-9-CM, International Classification of Diseases, 9^th^ Revision, Clinical Modification; LD, Legionnaires’ disease

^a^ For each patient, ICD-9-CM discharge diagnosis codes that were present in the 2 years prior to LD were not used for frequency calculations in the post-LD period. Each code remaining in the post-LD period was counted only once for each patient at first appearance in the medical record. The most frequent ICD-9-CM codes not present in every time period and for which about 10% of the cohort had the code are presented.

^b^ In the United States, the Federal Fiscal Year is October 1 to September 30.

^c^ The number of patients in each cumulative time period is based on the number of patients who survived to that time period and who had a hospitalization in the VA system up to the time period.

^d^ A review of the medical charts of patients with a LD discharge diagnosis code in the post-LD period indicated that, for all such patients (n=5), the code was assigned as a result of the original qualifying LD admission and not a new LD diagnosis.
