# Supplementary material for: Health after Legionnaires' disease: A description of hospitalizations up to 5 years after Legionella pneumonia
Source: PLoS One. 2021 Jan 11;16(1):e0245262. doi: 10.1371/journal.pone.0245262 (PMC7799844; doi:10.1371/journal.pone.0245262)
Supplement: S6 Table — (DOCX) [file pone.0245262.s006.docx]

S6 Table. Frequency of renal disease ICD-9-CM discharge diagnosis codes in Legionnaires’ disease (LD) patients at 6 months after LD and cumulative annually for five Federal Fiscal Years^a^, stratified by Intensive Care Unit (ICU) versus non-ICU admission during the LD hospitalization.

| **ICD-9-CM Code** | **Frequency of code (number and percent)** | | | | | | | | | | | |
| --- | --- | --- | --- | --- | --- | --- | --- | --- | --- | --- | --- | --- |
|  | **6 months** | | **1 year** | | **2 years** | | **3 years** | | **4 years** | | **5 years** | |
|  | ICU | Non-ICU | ICU | Non-ICU | ICU | Non-ICU | ICU | Non-ICU | ICU | Non-ICU | ICU | Non-ICU |
| **584.9^b^** | 5 | 3 | 6 | 3 | 6 | 4 | 9 | 5 | 10 | 8 | 12 | 10 |
|  | 63% | 38% | 67% | 33% | 60% | 40% | 64% | 36% | 56% | 44% | 55% | 45% |
| **403.90^c^** | 2 | 4 | 4 | 5 | 8 | 5 | 9 | 5 | 9 | 8 | 11 | 9 |
|  | 33% | 67% | 44% | 56% | 62% | 38% | 64% | 36% | 53% | 47% | 55% | 45% |
| **585.9^d^** | 2 | 2 | 3 | 3 | 7 | 4 | 8 | 4 | 8 | 7 | 8 | 7 |
|  | 50% | 50% | 50% | 50% | 64% | 36% | 67% | 33% | 53% | 47% | 53% | 47% |

^a^ In the United States, the Federal Fiscal Year is October 1 to September 30.

^b^ Acute renal failure, unspecified

^c^ Hypertensive chronic kidney disease

^d^ Chronic kidney disease, unspecified
